# First person – Nazeefa Nashrah

**DOI:** 10.1242/bio.062208

**Published:** 2025-09-03

## Abstract

First Person is a series of interviews with the first authors of a selection of papers published in Biology Open, helping researchers promote themselves alongside their papers. Nazeefa Nashrah is first author on ‘ Invasive goldfish (*Carassius auratus*) maintain aerobic scope across acute warm water temperatures’, published in BiO. Nazeefa is a prospective Master's student in the lab of Dr Nicholas Mandrak at the University of Toronto Scarborough, Canada, investigating the physiological responses of freshwater fish species to climate change, with a focus on metabolic performance.


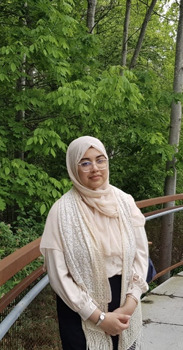


**Nazeefa Nashrah**

**Describe your scientific journey and your current research focus**

My journey in science began during my first year of university while pursuing my undergraduate degree. As part of a field-based lab assignment at the Toronto Zoo, we were asked to take photos and write about different animals. While observing the different species, I was particularly drawn to polar bears, as they are among the most threatened by global warming due to the rapid loss of Arctic Sea ice. This sparked my interest in marine conservation biology and motivated me to join Dr Mandrak's lab. Under the guidance of my lab mentors, I had the opportunity to work on several projects, including preparing eDNA samples and dissecting round goby and goldfish to determine their age, sex, and gut contents. I spent the summer and fall terms helping these projects, which became the highlights of my sophomore and junior years. Inspired by the lab's culture and research focus, I chose to pursue an honours thesis investigating the aerobic scope of invasive wild goldfish, co-supervised by Dr Melanie Massey and Dr Mandrak. For now, I'm excited to continue my studies with a focus on both ecophysiology and conservation biology.

**Who or what inspired you to become a scientist?**

My time working on the honours thesis revolved mainly around animal husbandry and respirometry trials, eventually followed by the preparation of the manuscript. As we wrapped up the trials, I could not let go of the thought in my mind that kept saying, ‘I will miss this so much’. It wasn't until the end of my final year that I realised just how much the lab had come to feel like home – I simply wasn't ready to say goodbye. I also want to acknowledge the incredible support I received throughout this journey. From the Mandrak lab members to even my mentors from other labs at UTSC, I was surrounded by a warm and encouraging community. With the unwavering support of both my advisors, I came to a clear realisation: I knew I wanted to stay in academia.

**How would you explain the main finding of your paper?**

Recently, there have been increasing concerns related to the spread of invasive wild goldfish in Canada. As average global water temperatures continue to rise because of climate change, with a rising likelihood of extreme heat waves, we wanted to see how wild goldfish respond to rapid changes in temperature. We looked to answer this by estimating the aerobic scope of wild goldfish. The aerobic scope is how much an animal can increase its energy above its normal resting state. What we found was that wild goldfish maintained stable aerobic scope under acute exposure (or short-term exposure) to temperatures of 26 and 30°C. This was very interesting because it suggests that the metabolic performance of wild goldfish is not compromised under increasingly acute exposure to hot temperatures, which wild goldfish frequently experience in their introduced environments.

this flexibility in thermal capacity could be very advantageous to wild goldfish as climate change warms up water temperatures, aiding in their invasive potential

**What are the potential implications of this finding for your field of research?**

Firstly, we get to know more about wild goldfish! Studies looking into goldfish before ours had worked with commercial goldfish, so our work aims to serve as a preliminary look at the metabolic responses of invasive goldfish in a local, wild population, linking aerobic metabolism to invasive species spread and impacts. In the context of invasion biology, our results suggest that even as maximum daily water temperatures increase, wild goldfish will be able to sustain the energy demands of important biological processes even at very high acute water temperatures. Most importantly, this flexibility in thermal capacity could be very advantageous to wild goldfish as climate change warms up water temperatures, aiding in their invasive potential.

**Figure:**
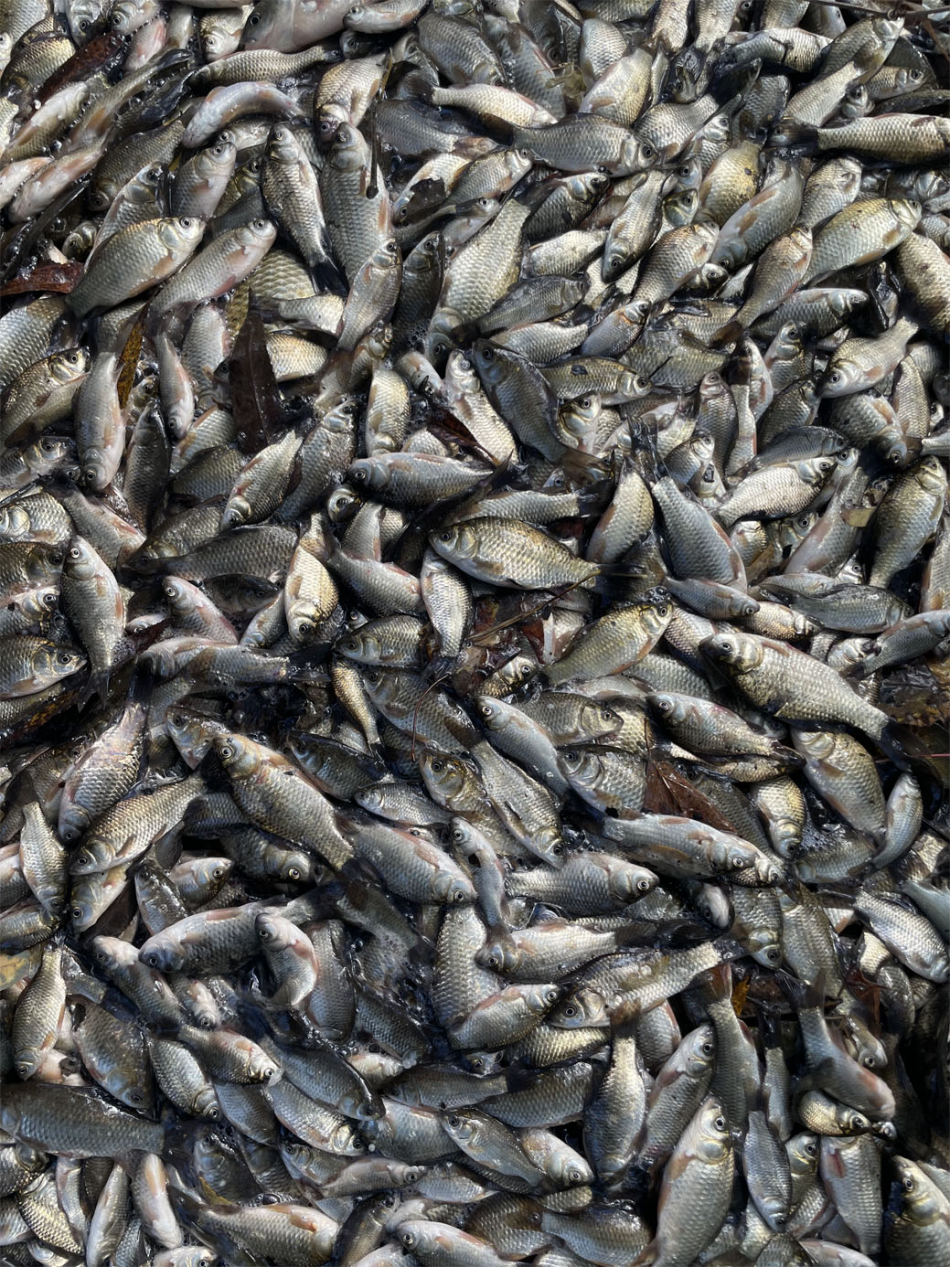
**In November of 2024, the Mandrak lab caught approximately 1000-1500 wild goldfish from Grindstone Creek, Hamilton, Ontario, Canada.** Picture credit: Dr Melanie D. Massey.

**Which part of this research project was the most rewarding?**

One of the most rewarding parts of our project was being able to execute the respirometry trials even with a relatively short timeline and logistical difficulties. It truly made me appreciate the complexity that rests in the data collection involved in physiology experiments, but most importantly, it reminded me of how important perseverance is in science. I also had the opportunity to present our findings at the undergraduate research day at UTSC, which was very rewarding as we not only received positive feedback from faculty, but I got to have interesting and meaningful conversations with my colleagues. Ultimately, the most rewarding part has to be when our manuscript was accepted! I am so excited for everyone to see it.

**What do you enjoy most about being an early-career researcher?**

I feel like, as an early-career researcher, I have so many opportunities to learn, even from a simple meeting with my PI. I am also provided with direct mentorship and coupling that with the ability to learn from passionate scientists, I can see my future self getting so much room to grow academically, which excites me the most.

**What piece of advice would you give to the next generation of researchers?**

Although hardships are inevitable, they are often followed by success/rewards – so keep persevering. No matter what stage of your career you're in, challenges will arise, and they can be difficult to handle, especially on a mental and emotional level. But every obstacle is an opportunity for growth so stay resilient and never give up.

**What's next for you?**

For now, I am looking forward to developing my next research idea for a Master's!

## References

[BIO062208C1] Nashrah, N. A., Mandrak, N. E. and Massey, M. D. (2025). Invasive goldfish (*Carassius auratus*) maintain aerobic scope across acute warm water temperatures. *Biol. Open*, 14. bio062160. 10.1242/bio.062160 40767447 PMC12444859

